# Genetic Interactions of STAT3 and Anticancer Drug Development

**DOI:** 10.3390/cancers6010494

**Published:** 2014-03-06

**Authors:** Bingliang Fang

**Affiliations:** Department of Thoracic and Cardiovascular Surgery, The University of Texas MD Anderson Cancer Center, Houston, TX 77030, USA; E-Mail: bfang@mdanderson.org; Tel.: +1-713-563-9147; Fax: +1-713-794-4901

**Keywords:** genetic interaction, cancer, drug development, STAT3, Ras, EGFR, redox, reactive oxygen species, synthetic lethality

## Abstract

Signal transducer and activator of transcription 3 (STAT3) plays critical roles in tumorigenesis and malignant evolution and has been intensively studied as a therapeutic target for cancer. A number of STAT3 inhibitors have been evaluated for their antitumor activity *in vitro* and *in vivo* in experimental tumor models and several approved therapeutic agents have been reported to function as STAT3 inhibitors. Nevertheless, most STAT3 inhibitors have yet to be translated to clinical evaluation for cancer treatment, presumably because of pharmacokinetic, efficacy, and safety issues. In fact, a major cause of failure of anticancer drug development is lack of efficacy. Genetic interactions among various cancer-related pathways often provide redundant input from parallel and/or cooperative pathways that drives and maintains survival environments for cancer cells, leading to low efficacy of single-target agents. Exploiting genetic interactions of STAT3 with other cancer-related pathways may provide molecular insight into mechanisms of cancer resistance to pathway-targeted therapies and strategies for development of more effective anticancer agents and treatment regimens. This review focuses on functional regulation of STAT3 activity; possible interactions of the STAT3, RAS, epidermal growth factor receptor, and reduction-oxidation pathways; and molecular mechanisms that modulate therapeutic efficacies of STAT3 inhibitors.

## 1. Introduction

Signal transducer and activator of transcription 3 (STAT3) is known to promote tumor cell proliferation, survival, and invasion [1,2], mediate procarcinogenic inflammation while suppressing the host’s antitumor immunity [3], induce cancer stem cell renewal [4,5,6], enhance epithelial-mesenchymal transition [7,8] and angiogenesis [9,10], and generate positive autocrine and paracrine feedback loops between tumor cells and their microenvironments [11,12] by regulating expression of a number of cancer-related key proteins, cytokines, and growth factors. Ectopic expression of constitutive STAT3 is sufficient to induce transformation of rodent cells *in vitro* and tumor formation *in vivo* [3,13]. Constitutive activation of STAT3 has been reported in many human cancer cell lines and primary tumors, and this activation is associated with poor outcomes of a number of cancers. Inhibiting STAT3 expression or phosphorylation using antisense oligonucleotides and small-molecule inhibitors suppressed the growth of human and murine tumors in animal models [14,15,16], demonstrating that STAT3 is a potential target for cancer therapy. Substantial efforts have been devoted to developing strategies for pharmaceutical intervention directed toward STAT3 functions, including interrupting STAT3 dimerization and inhibiting its interaction with its upstream activating kinases or downstream DNA targets using oligonucleotides [17], peptides [18], and small-molecule inhibitors [19,20,21,22]. A number of STAT3 inhibitors have been identified and evaluated their antitumor activity *in vitro* and *in vivo* in experimental tumor models [23,24,25,26]. Moreover, several U.S. Food and Drug Administration (FDA)-approved therapeutic agents are reported to function as STAT3 inhibitors. For example, pyrimethamine, an antimalarial drug [27,28], inhibits STAT3 phosphorylation and is in clinical investigation for treatment of leukemia [26,29]. In addition, sorafenib, an inhibitor of RAF and multiple other kinases [30,31] approved for the treatment of advanced renal and liver cancer, inhibits STAT3 phosphorylation, possibly by activating the phosphatase shatterproof 2 (SHP2), as knockdown of SHP2 expression inhibited sorafenib-induced STAT3 phosphorylated Y705 (pY705) dephosphorylation [32,33]. Arsenic trioxide, an inorganic compound used to treat leukemia, inhibits STAT3 phosphorylation possibly by inhibiting its upstream kinases [34,35]. Furthermore, auranofin, a thioredoxin inhibitor that is used to treat rheumatoid arthritis [36], inhibits Janus kinase 1 (JAK1)/STAT3 phosphorylation [37,38]. However, most STAT3 inhibitors have yet to be translated to clinical trials for cancer treatment, presumably because of pharmacokinetic, efficacy, and safety issues. 

Lack of therapeutic efficacy may be caused by low potency of the candidate drug in inhibiting its proposed target. Nevertheless, mutation analyses of primary cancer cells for genes encoding kinases or known to be associated with cancers have revealed that individual tumors may harbor multiple changes in such genes [39,40,41,42]. Several important signaling pathways are often cooperatively involved in tumorigenesis and malignant evolution of cancers [39,40,41,42,43]. As a result, interrupting just one of these pathways is often insufficient to induce cancer cell death in most cases because redundant input from different pathways drives and maintains downstream signaling, leading to low therapeutic efficacy because of inhibition of a single target [44,45]. Conceivably, agents that can modulate the functions of multiple cancer-related targets and/or pathways will improve the efficacy of cancer therapy because they are more likely to have a broad anticancer spectrum and less likely to induce therapy resistance than single-target anticancer agents. Indeed, multitarget agents such as sorafenib and sunitinib, which block several kinases, have proven to be useful clinically for cancer treatment and have a broader spectrum of activity than single-target agents such as erlotinib and gefitinib [46]. The knowledge on genetic interactions among cancer-associated pathways may facilitate development of multitarget agents or rational design of combinatorial therapy using single-target agents to enhance therapeutic efficacy. This review describes potential interactions of STAT3 with other cancer-associated pathways and molecular mechanisms that modulate therapeutic efficacies of STAT3 inhibitors.

## 2. STAT3-Associated Single-Gene Diseases

The human STAT3 gene is located on chromosome 17q21.31 and encodes two major isoforms of STAT3 proteins via alternative mRNA splicing: STAT3α (p92) and STAT3β (p83). A 55-residue *C*-terminal transactivation domain of STAT3α is deleted in STAT3β and replaced by seven unique *C-*terminal residues (CT7) whose functions remain undefined [47]. Both isoforms contain STAT protein interaction, DNA-binding, and Src homology 2 (SH2) domains, but only STAT3α contains a transactivation domain at the *C*-terminus. 

Targeted disruption of the murine Stat3 gene leads to early embryonic lethality. Stat3-deficient embryos have exhibited rapid degeneration from embryonic day 6.5 to embryonic day 7.5 [48]. Ablation of isoform-specific gene expression demonstrated that Stat3β is not required for viability of mice but is involved in inflammation because mice with deficiency of Stat3β were viable and fertile; in contrast, Stat3α-deficient mice died within 24 h after birth [49]. In comparison with Stat3^−/−^ mice that die at early stages of embryonic development [48], expression of Stat3β rescues the embryonic lethality of a Stat3-null mutation, and Stat3β alone can induce transient transcription of acute-phase genes, suggesting that although Stat3β does not have a transactivation domain, it may induce the expression of specific Stat3 target genes by interacting with other transcriptional factors. A study of green fluorescent protein-tagged Stat3α and Stat3β demonstrated that the two isoforms have distinct intracellular dynamics, with Stat3β exhibiting prolonged nuclear retention and reduced intranuclear mobility, especially following ligand stimulation, and prolonged nuclear retention but not reduced intranuclear mobility mapping to the CT7 domain of Stat3β [50].

*De novo* dominant-negative mutations in the DNA-binding domain of STAT3 have been identified in autosomal dominant or sporadic cases of hyperimmunoglobulinemia syndrome (HIES; or Job syndrome) in humans [51]. These mutations rendered patients’ peripheral blood cells defective in responding to interleukin (IL)-6 and IL-10 stimulation. *De novo* deficiency mutations of STAT3 have also occurred in the SH2 domain [52,53] and the transactivation domain [53,54] of STAT3 in patients with HIES from different ethnic groups [55]. These dominant-negative mutations in STAT3 impaired the development of IL-17-producing T cells, which is critical to the clearance of fungal and extracellular bacterial infections and may be the underlying mechanism of susceptibility to recurrent infections commonly seen in HIES patients [56,57,58].

There are only a few reports of STAT3 gene mutation in human cancer cells. In one report, a patient with HIES owing to STAT3 mutation had a subsequent primary parotid gland diffuse large B-cell lymphoma [59]. Because HIES patients are predisposed to lymphoma [60], STAT3 mutations may occur with other types of lymphoma in HIES patients. Mutations in the SH2 domain that lead to constitutive activation of STAT3 were recently reported in patients with human inflammatory hepatocellular adenoma, a benign liver tumor, suggesting that STAT3-activating mutations play roles in human tumorigenesis [61]. In contrast with STAT3 gene mutations, constitutive activation of STAT3 and/or STAT5 at the protein level has occurred in many human cancer cell lines and primary tumors. For example, persistent activation of STAT3 and STAT5 has been reported in breast cancer, lung cancer [62], glioma [63], liver cancer [15], pancreatic cancer [64,65], and nasopharyngeal carcinoma [66] cases. STAT3 also is activated in 77% of lymph node metastases and 67% of bone metastases of prostate cancer [67]. In addition, constitutive JAK3 and STAT5 activation has been observed in patients with T-cell leukemia caused by human T-cell leukemia virus type 1 [68]. High levels of STAT3 protein expression have been associated with poor tumor differentiation and/or development of metastasis and poor survival rates in leukemia [69], lymphoma [70], osteosarcoma [71,72], glioma [63], gastric adenocarcinoma [73,74], colorectal cancer [75,76], bladder cancer [77], and cervical squamous cell carcinoma [78] cases. These data demonstrate the critical roles of the STAT3 pathway in malignant progression.

## 3. Genetic Interactions and STAT3 Activation

**Figure 1:**
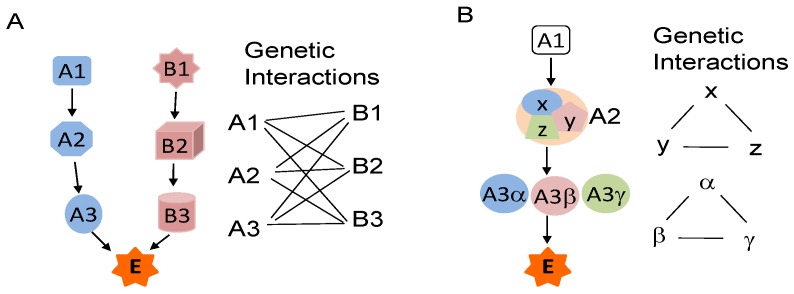
Diagram of genetic interactions. (**A**) The essential biological function E is regulated by pathways A and B. A functional change in either of these pathways, such as a mutation in A1 or B1, is insufficient to induce dysfunction of E. However, the simultaneous presence of a mutation in A1 and a mutation in any of B1, B2, or B3 induces dysfunction of E (or phenotype changes). Thus, A1 has genetic interaction with B1, B2 and B3, and vice versa; (**B**) The essential biological function E is regulated by pathway A alone, in which A2 is a multiprotein complex composed of X, Y, and Z, while A3 has homologues of α, β and γ. Genetic interaction may exist among X, Y and Z, and among A3α, β and γ.

Genetic interactions are functional cross-talks among genes, which regulate or compensate for one another in many signaling and/or metabolic pathways, leading to phenotypic changes, including disease status (synthetic sickness) and viability (synthetic lethality or semilethality) alterations. Genetic interactions have been used by investigators to identify genes that are crucial to the survival of certain oncogene-transformed cells [79,80,81,82] or that sensitize cells to chemotherapy [83,84] or to find small molecules that selectively induce the death of oncogene-transformed isogenic cells [85,86,87]. Several models have been proposed to account for these genetic interactions [88,89,90], including the components of parallel pathways that combine to regulate an essential biological function, subunits of an essential multiprotein complex, or components of a linear essential pathway (Figure 1) [91]. Thus, key components in the signaling pathways that regulate STAT3 functions or regulate parallel pathways involved in similar biological processes or common downstream targets as STAT3 may be partners of genetic interactions with STAT3. The status and functionality of these partners are critical determinants of cellular fate when the functionality of STAT3 is disrupted.

**Figure 2:**
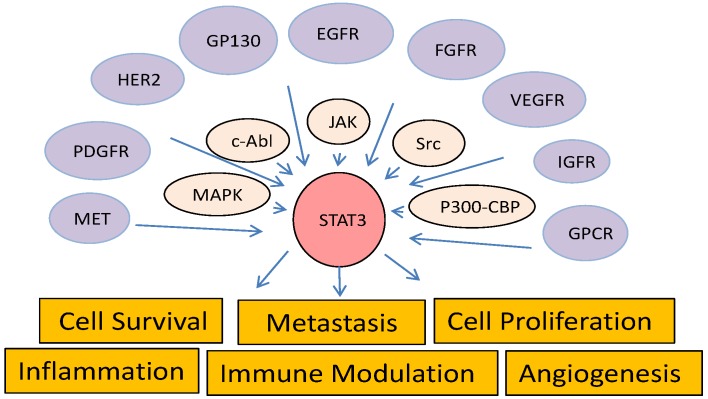
Diagram of STAT3 pathways. STAT3 is activated by upstream receptor tyrosine kinases, intracellular kinases, or histone acetyltransferases and regulates a diverse biological functions.

STAT3 is activated by tyrosine phosphorylation in response to stimulation of a variety of cytokines, such as IL-6, leptin, prolactin, erythropoietin, and thrombopoietin, and growth factors, such as epidermal growth factor (EGF), fibroblast growth factor, insulin-like growth factor, hepatocyte growth factor, platelet-derived growth factor (PDGF), and vascular endothelial growth factor (VEGF) [92,93,94,95,96]. Interaction of those ligands with their receptors in a cell triggers receptor phosphorylation by intrinsic receptor tyrosine kinases or non-receptor protein tyrosine kinases such as JAK and Src family members, resulting in translocation of STAT3 protein from the cytoplasm to the phosphorylated receptors and further STAT3 phosphorylation at Y705 by these kinases (Figure 2). Once phosphorylated, STAT3 forms dimers and translocates to the nucleus, where it activates expression of its target genes [97,98]. In addition, STAT3 has been identified in mitochondria [99] and some endosomes in the cytoplasm [100]. Mitochondrial localized STAT3 was independent of Y705 phosphorylation and DNA-binding activity but required for Ras-mediated oncogenic transformation. In contrast, enrichment of STAT3 in some endosomes is stimulated by IL-6 and dependent on pY705, co-localized with myeloid differentiation primary response gene 88 (MYD88), and enhanced by dominant-negative mutants of GTPase dynamin II [100]. Interestingly, a gain-of-function mutation of MYD88 that promotes cell survival via activation of IL-1 receptor-associated kinase 1, nuclear factor (NF)-κB, and JAK/STAT3 signaling is frequently found in cases of a subtype of B-cell lymphoma [101]. Knockdown of MYD88 expression has significantly inhibited secretion of IL-6 and phosphorylation of STAT3 in B-cell lymphoma cells.

The activity of STAT3 is also regulated by serine phosphorylation and lysine acetylation. Phosphorylation of S727 in STAT3 by c-Jun *N*-terminal kinase (JNK) and extracellular signal-regulated kinase (ERK) may enhance or be required for STAT3/DNA interaction [102,103] and promote tumorigenesis independently of pY705 [102,104]. Blockage of S727 phosphorylation via substitution of the this serine with an alanine led to death soon after birth or growth retardation in transgenic mice [105]. Nevertheless, S727 phosphorylation also has negatively regulated Y705 phosphorylation [106,107]. Acetylation of K685 in STAT3 by CREB-binding protein/p300 is stimulated by IL-6 and interferon-α independently of Y705 and S727 phosphorylation and is required for STAT3 activation and nuclear accumulation [108,109]. This acetylation also mediates cross-talk between the STAT3 and NF-κB pathways [110]. A recent study demonstrated that multiple lysine-acetylation sites adjacent to Y705 are essential for pY705 phosphorylation in STAT3 [111].

Within cells, STAT3 activity is also regulated by several inhibitory molecules that prevent its continued activation. For example, tyrosine dephosphorylation of receptors and JAK receptor-associated phosphatases lead to inactivation of JAKs and prevent further STAT activation [112,113,114]. Histone deacetylases and NAD-dependent deacetylase sirtuin-1 inactivate STAT3 by reversing lysine acetylation [109,111,115]. Moreover, expression of STAT3-activated suppressor of cytokine signaling (SOCS) proteins [116,117] or cytokine-induced SH2 protein [118] provides negative feedback for JAK/STAT activation. SOCS proteins bind directly to and inhibit the activity of tyrosine-phosphorylated JAKs and cytokine receptors. Also, phosphorylated STAT proteins can be inactivated in the nucleus via dephosphorylation [119] or by interaction with the nuclear protein inhibitor of activated STAT [120,121] or cytoplasmic protein aplasia Ras homolog member I [122]. The latter protein was found to form a complex specifically with STAT3 in the cytoplasm, preventing IL-6-induced STAT3 accumulation in the nucleus and inhibiting STAT3-dependent promoter activity while only moderately affecting STAT3 phosphorylation [122]. In addition, tripartite motif 8 (TRIM8) interacts with protein inhibitor of activated STAT3 (PIAS3), which inhibits IL-6-ependent activation of STAT3. Ectopic expression of TRIM8 cancels the negative effect of PIAS3 on STAT3 activation via either degradation of PIAS3 in the ubiquitin/proteasome pathway or exclusion of PIAS3 from the nucleus.

The complexity of STAT3 activity regulation networks suggests that pharmaceutical inhibition of STAT3 activity can be achieved with a variety of mechanisms. Small-molecule inhibitors of upstream kinases JAK, Src, and Bcr-Abl are predicted to inhibit STAT3 activation. Indeed, the JAK inhibitors INCB16562, AZD1480, and tofacitinib (CP-690550) have potently blocked STAT3 signaling, suppressed cancer cell proliferation, or induced apoptosis *in vitro* and tumor growth *in vivo* [123,124,125]. Additionally, the JAK inhibitors ruxolitinib and tofacitinib have been approved for treatment of myelofibrosis [126,127,128] and rheumatoid arthritis [129,130], respectively. However, depletion of c-Src by small interfering RNA (siRNA) and sustained inhibition of Src by dasatinib led to JAK-dependent STAT3 activation in lung cancer cells *in vitro* and *in vivo* [131]. Phosphorylated STAT3 levels were initially decreased but strongly increased after sustained treatment with dasatinib, suggesting the existence of a compensatory feedback pathway that supports cancer cell survival by regulating STAT3 activity [132].

## 4. Cross-Talk of STAT3 with Other Cancer-Associated Pathways

### 4.1. RAS Pathway

As members of a subfamily of small guanine nucleotide-binding proteins, Ras proteins (HRAS, KRAS, and NRAS) cycle between active GTP-bound and inactive GDP-bound forms [133,134]. Binding of Ras with GTP is facilitated by guanine nucleotide exchange factors (GEFs) via catalysis of the release of GDP and is required for the interaction of Ras with target proteins [135]. Intrinsic GTPase activity enhanced by GTPase-activating proteins [136] converts GTP to GDP, leading to inactive GDP-bound Ras. Ras mutations that diminish GTPase activity or decrease GDP-binding capacity render Ras in a constitutively active GTP-bound status. Activating mutations in RAS genes are among the most frequently observed oncogenic mutations in human cancer cases. In the absence of a Ras mutation, increased Ras activity in human cancer cells frequently results from gene amplification [137,138], gene overexpression [139], or an increase in activity of upstream signals from tyrosine kinase growth factor receptors such as Her2 and EGF receptor (EGFR) [140,141]. Activation of EGFR results in the assembly of Grb2 and the Son of Sevenless (SOS) complex; SOS is one of the guanine nucleotide exchange factors that activate RAS [141,142]. RAS activation has resulted in stimulation of a wide range of downstream signaling pathways, most notably the RAF/mitogen-activated protein kinase (MAPK) kinase (MEK)/ERK [143,144] and phosphoinositide 3-kinase (PI3K)/AKT/mammalian target of rapamycin pathways. GTP-RAS binds directly to and activates the catalytic subunit of PI3K p110 independently of the regulatory subunit PI3K p85 [145,146]. 

**Figure 3:**
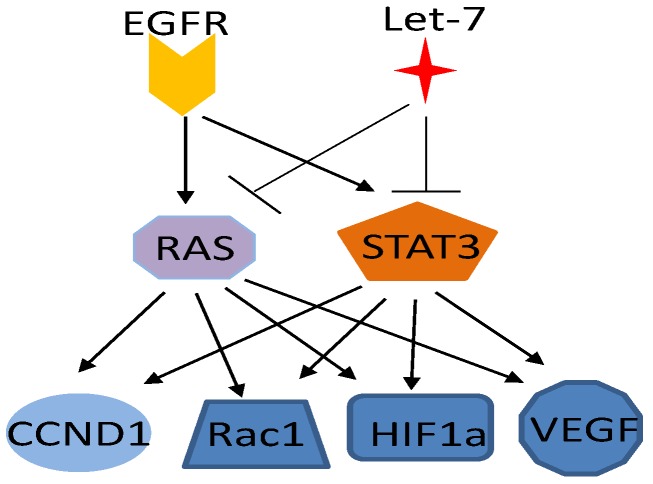
Ras and STAT3 mediated parallel pathways for EGFR and let-7. Ras and STAT3 has common upstream regulators and downstream targets.

Both Ras and STAT3 proteins are activated by EGFR [96,141,147] and suppressed by the microRNA let-7 [148,149,150,151], and both of them regulate the common downstream targets, such as cyclin D1 [152,153,154], Bcl2 family proteins [155,156,157,158], Rho family GTPases [159,160,161], hypoxia-inducible factor-1α [162,163], and VEGF [9,164], suggesting that Ras and STAT3 mediate certain parallel, complementary, or coordinated biological processes (Figure 3). In fact, STAT3 has been found to be an important factor in Ras-mediated oncogenic transformation. Ras transduction in different cell types has induced secretion of the cytokine IL-6, whereas knockdown of IL-6 expression, genetic ablation of the IL-6 gene, and treatment with a neutralizing anti-IL-6 antibody has retarded Ras-driven tumorigenesis [65,165]. A recent study demonstrated that mitochondrial STAT3 is required for Ras-induced malignant transformation [99]. This function of STAT3 may be involved in glucose and energy metabolism and does not require Y705 phosphorylation or the presence of intact SH2 and DNA-binding domains but does require S727 phosphorylation. 

Studies of K-ras-transgenic mice demonstrated that Stat3 phosphorylation occurs in tumors at multiple stages of K-ras-induced pancreatic tumorigenesis but not in normal pancreatic tissue [65,166]. Disruption of the IL-6 gene and conditional inactivation of STAT3 reduced progressive pancreatic intraepithelial neoplasia (PanIn) lesion and pancreatic ductal adenocarcinoma formation in mutant K-ras-transgenic mice [65,166], suggesting that STAT3 activity is required for the development of the earliest premalignant pancreatic lesions, acinar-to-ductal metaplasia, and PanIN and for the progression from PanIN to invasive pancreatic ductal adenocarcinoma [65]. Disruption of Socs3, an endogenous feedback inhibitor of the STAT3 signaling pathway, in mutant K-ras-transgenic mice resulted in early, robust activation of STAT3 and high-grade PanIn lesions [166]. Also, Ras- and Rac1-mediated p38 and JNK signals are required for STAT3 transcriptional activity induced by the Src oncoprotein [167]. STAT3-mediated gene regulation by v-Src is strictly Ras-dependent in NIH 3T3 cells, as STAT3 function is completely abrogated by expression of dominant-negative Ras or Rac1.

Both Ras and STAT3 regulate the activity of Rho-family small GTPases. STAT3 regulates Rac1 activity by interacting with the Rac1 activator betaPIX in the cytoplasm, thereby modulating the organization of the actin cytoskeleton and cell migration [159]. Although evidence of Rho GTPase-mediated Ras activation is lacking, researchers have shown that Rho GTPases such as RhoA, Rac1, and Cdc42 can regulate STAT3 phosphorylation and nuclear translocation [168]. Rho GTPases are required for G protein-coupled receptor (GPCR)-mediated JAK/STAT signaling [169]. GPCR-stimulated Rac activation resulted in generation of reactive oxygen species (ROS), which in turn activated the JAK/STAT pathway. Rho GTPase can also activate STAT3 through direct interaction with it [170], via the IL-6 autocrine pathway [171,172] or Rho-associated kinase [173]. STAT3 mediates RhoA-induced NF-κB and cyclin D1 expression and NF-κB nuclear translocation [173]. In addition, Rac1 is a key mediator of IL-6/gp130-induced STAT3 S727 phosphorylation by SEK-1/MKK-4 [174].

The interaction between STAT3 and Ras signaling pathways suggests that the functional status of STAT3 affects responses of cancer to treatment with agents targeting these signaling pathways. Indeed, a study of response to treatment with the MEK inhibitor AZD6244 using a panel of lung cancer cell lines revealed that activation of the STAT3 pathway is associated with resistance to AZD6244 [175]. Inhibiting STAT3 activity with siRNA or a small-molecule inhibitor dramatically sensitized lung cancer cells to treatment with AZD6244 both *in vitro* and *in vivo*. Similarly, investigators observed a synergistic effect between a PI3K inhibitor and a STAT3 inhibitor in human gastric cancer cells harboring KRAS mutations [176]. Moreover, small molecules that induced synthetic lethality in KRAS mutant cancer cells [87,177] were found to be a novel class of STAT3 inhibitors [178,179], demonstrating that genetic interaction between Ras and STAT3 pathways can be explored for genotype-specific anticancer therapeutics.

### 4.2. EGFR Pathway

EGFR-mediated signaling pathways are known to be a driving force in lung tumorigenesis and have been extensively investigated as targets for cancer therapy. Activating EGFR mutations are detected in about 10%–17% of human lung adenocarcinoma cases, with higher percentages in women and patients with no smoking history [39,180,181,182]. In addition, EGFR gene amplification and overexpression have been reported in 20%–60% of primary non-small cell lung cancer tumors [183,184]. Amplification of the EGFR gene has been observed in colorectal cancer [185,186], pancreatic cancer [187,188], head and neck cancer [189], and glioma [190] cases. Inducible expression of human lung cancer-related mutant EGFR genes in transgenic mice caused the development of lung adenocarcinoma, whereas stopping inducible expression of the mutant EGFR genes led to lung tumor regression [191,192], demonstrating that activating EGFR mutations are required and sufficient for lung cancer tumorigenesis and malignancy maintenance. Small-molecule inhibitors (erlotinib, gefitinib, and afatinib) and a monoclonal antibody (cetuximab) targeted to EGFR have been used for treatment of lung, colorectal, head and neck, and pancreatic cancers.

STAT3 is identified because of its activation by tyrosine phosphorylation in response to exposure to EGF and IL-6 [96]. Activation mutations of EGFR have been reported to activate STAT3, which is required for the oncogenic effects of EGFR mutation**s** [193,194]. EGFR can activate STAT3 via direct recruitment and activation of STAT3 [195], upregulation of IL-6 expression, and activation of the gp130/JAK/STAT3 pathway [194] or activation of STAT3 by activating non-receptor tyrosine kinases such as Src and Pyk2 [196,197]. An *in vitro* study using recombinant proteins demonstrated that STAT3α and STAT3β formed stable complexes with EGFR and were phosphorylated in tyrosine by the EGFR and activated for binding to DNA [198]. Activation of EGFR results in autophosphorylation of several tyrosine residues in EGFR, which provide docking sites for direct recruitment of downstream substrates. In EGFR, pY1068 and pY1086 are the docking sites for STAT3 [195] and Grb2, an SH2 domain-containing adaptor protein [199,200]. In contrast, Shc, another SH2 domain-containing adaptor protein, binds to Y1148 and Y1173 in EGFR [190,201], whereas phospholipase Cγ binds to Y992 [202] and the protein tyrosine phosphatase SHP-1 binds to Y1173 in EGFR [203].

As a key downstream mediator of the EGFR signaling pathways, STAT3 is crucial to EGFR-mediated cell growth *in vitro*. Inhibition of STAT3 expression by an antisense oligonucleotide dramatically suppressed the transforming growth factor-α/EGFR-mediated growth of transformed epithelial cells [204]. STAT3 is critically involved in EGFR-induced cancer cell migration and invasion [205,206]. Moreover, EGFR physically interacts with STAT3 in the nucleus, leading to transcriptional activation of inducible nitric oxide synthase [207], suggesting that STAT3 functions not only as a downstream mediator of EGFR but also as a partner of EGFR in regulating diverse biological functions. In addition to EGFR, STAT3 is activated by many other growth factor receptors, such as PDGF receptor [208,209] and MET [210,211] (Figure 2). STAT3 may be a mediator of MET-induced resistance to anti-EGFR therapy. In cancer cells with high MET activity, inhibition of EGFR activity alone likely is not sufficient to abrogate STAT3 activity. Synthetic lethal screening of an EGFR-centered signaling network using a siRNA library revealed that STAT3 is one of the targets that synergize with EGFR antagonists to reduce cancer cell viability and tumor size [84]. Inhibition of the STAT3 pathway has been shown to overcome resistance of lung cancer [212,213], head and neck cancer [214], pancreatic cancer [215], and glioma [216] to anti-EGFR therapy. Furthermore, treatment of cancer cells with EGFR inhibitors such as afatinib and dacomitinib can activate the IL-6/JAK/STAT3 signaling pathway, which in turn induces resistance to these agents [217]. Inhibiting IL-6/JAK/STAT3 signaling or STAT3 activity alone with antisense oligonucleotides, siRNA, or small-molecule inhibitors can dramatically sensitize cancer cells to treatment with EGFR inhibitors [212,214,217].

### 4.3. Reduction-Oxidation Pathways

In reduction-oxidation (redox) reactions, a molecule’s oxidation state changes because of a gain or loss of electrons. Inside a cell, redox metabolism is balanced by production and elimination of ROS, a group of oxygen- or nitrogen-containing molecules that are highly active in redox reactions. Produced in living organisms via a wide range of physiological process, ROS can serve as second messengers in response to exposure to growth factors, hormones, and cytokines. For example, H_2_O_2_ participates in essentially all receptor tyrosine kinase-mediated signal transduction, including EGF, PDGF, insulin, and cytokines [218,219]. Also, ligand-receptor interactions can induce the production of ROS, which regulates the intracellular activity of key signaling components, including protein kinases and protein phosphatases [218], and is required for cell proliferation [220], adhesion [221], migration [222,223], differentiation [224], oncogenic transformation [225], and apoptosis [226,227,228]. ROS also are involved in several critical steps in cancer initiation and progression, including somatic mutations and genome instability in cancer cells [229,230,231], epithelial-mesenchymal transition [230,232], metastasis [233,234,235], angiogenesis [236,237], and maintenance of stem cell status [238,239]. Nevertheless, deregulation of redox metabolism in cancer cells caused by overexpression of oncogenes such as *Ras* [225], *c-Myc* [231], *c-Abl* [240], and *Src* [241] and growth factor receptors such as c-MET [230], insulin-like growth factor receptor [242], EGFR [243], and VEGF receptor [244] may render cancer cells more vulnerable than normal cells to oxidative stress-induced death [245,246]. Indeed, ROS generation and consequent oxidative damage are among the major mechanisms by which radiotherapy [247,248] and chemotherapy [249] induce apoptosis.

Cellular ROS may have either a stimulatory or inhibitory effect on STAT3 signaling depending on the cellular context or level or duration of ROS generation in a cell. Oxidative stress has been reported to stimulate the JAK/STAT pathway [250,251,252]. Also, H_2_O_2_ stimulates the activity of the STAT kinases JAK2 and TYK2 and activates STAT1 and STAT3 in fibroblasts, lymphocytes, and cancer cells. Activation of STATs by PDGF is markedly inhibited by the ROS scavenger *N*-acetyl-L-cysteine and diphenylene iodonium, indicating that ROS production contributes to STAT activation in response to PDGF exposure. These findings demonstrate that the JAK/STAT pathway responds to intracellular ROS induction and that PDGF uses ROS as second messengers to regulate STAT activation [250]. Stimulation of the JAK/STAT cascade by angiotensin II requires O_2_^−^ anions generated by the NAD(P)H oxidase system [253]. ROS are mediators of GPCR-stimulated Rac activity and subsequent activation of the JAK/STAT pathway [169]. Increasing evidence suggests that the cellular redox state is involved in regulating tyrosine phosphatase activity via reversible oxidization of catalytic cysteine to sulfenic acid (Cys–SOH) [254,255,256]. Moreover, cell death caused by oxidative stress triggers secretion of IL-11 from dying cells because of ERK2-mediated activation of the transcriptional factor Fra-1, leading to STAT3 activation and compensatory proliferation of neighboring cells [257]. Interestingly, ROS do not induce tyrosine phosphorylation of STAT3 in SYK-deficient human leukemia cells. Inhibition of SYK activity by a small molecule prevents ROS-induced activation of STAT3 and overcomes the resistance of human B-lineage leukemia and lymphoma cells to ROS-induced apoptosis [258], indicating that SYK plays an indispensable role in oxidative stress-induced STAT3 activation in B-cell leukemia and lymphoma cells.

Evidence also suggests that ROS attenuate cytokine-induced JAK/STAT activation [259,260,261]. Four cysteine residues in the catalytic domain of JAK2 (Cys866, Cys917, Cys1094, and Cys1105) provide a mechanism for redox regulation in JAK2 via oxidation and reduction of these residues [262]. JAK2 is catalytically inactive when oxidized; its activity can be restored via reduction to the thiol state. In addition, nitric oxide can markedly suppress endogenous tyrosine phosphorylation of JAK3 and STAT5 [257] and leptin-mediated activation of STAT3 [260]. Nitric oxide and other thiol oxidants can inhibit the autokinase activity of murine JAK2 *in vitro*, presumably via oxidation of crucial dithiols to disulfides in JAK2. The autokinase activity of JAK3 responds in a similar fashion to exposure to thiol redox reagents *in vitro* and nitric oxide donors *in vivo*. Treatment with parthenolide, an anti-inflammatory compound, increases intracellular ROS level and inhibits JAK1 and STAT3 activation but stimulates the MAPK pathways. Pretreatment with the antioxidant *N*-acetyl-L-cysteine completely suppressed the inhibitory effect of parthenolide on JAK1 and STAT3 [259]. The cysteine residues in STAT3 can be modified by S-glutathionylation in response to mild oxidative stress, attenuating IL-6-mediated STAT3 activation, as glutathionylated STAT3 is a poor substrate for JAKs [263].

In contrast to many reports on ROS-mediated changes in STAT3 activity, only a few reported studies have examined regulation of ROS levels by STAT3. In cardiomyocytes, constitutively active STAT3 protects against hypoxia- and/or reoxygenation-induced injury by scavenging ROS via upregulation of expression of manganese superoxide dismutase and its enzyme activity [264] and via upregulation of expression of the ROS scavengers metallothioneins [265]. Depletion of mitochondrial STAT3 has resulted in decreased ATP production and triggered ROS production [266,267]. This cross-talk between STAT3 and redox pathways suggests that STAT3 plays a critical role in oxidative stress-mediated cancer therapy. In fact, the STAT3 inhibitors developed in our laboratory [178,179] drastically induced ROS generation in the susceptible cancer cells [179,268,269]. Scavenging of ROS using antioxidants such as nordihydroguaiaretic acid, aesculetin, baicalein, caffeic acid, and flavonoids abolished their apoptosis-induction activity [268,269], demonstrating the critical roles of oxidative stress in antitumor activity induced by this class of STAT3 inhibitors.

## 5. Conclusions and Perspectives

Efficacy and toxicity are the two major issues in drug development. A survey of anticancer agents evaluated in human studies from 1991 to 2000 demonstrated that only about 5% of those entering clinical trials were approved for clinical use, and the majority of treatment failures in late-phase clinical trials of candidate anticancer agents resulted from a lack of efficacy of the drugs [270,271,272,273]. Genetic interactions among pathways cooperatively involved in initiation and maintenance of malignancy indicate that the therapeutic efficacy of single-target therapy for cancer is highly dependent on the cellular context of signaling networks. Single-target therapy may be highly effective for cancers that are addicted to certain oncogenes. For example, treatment of various cancers in humans with the EGFR inhibitors erlotinib and gefitinib [274] and the BCR-Abl inhibitor imatinib [275] already has been successful. Nevertheless, the success of such single-target therapeutics relies on the identification of potential responders in patient populations. The use of EGFR mutations as biomarkers to identify responders has greatly contributed to the success of gefitinib [181,274,276] and erlotinib [181], because both gefitinib [277] and erlotinib [278,279] failed to be beneficial in randomized phase 3 trials in unselected patient populations.

The advances in knowledge about networks of genetic interactions are expected to impact strategies for enhancing the therapeutic efficacy of anticancer therapy. The interactions of the STAT3 pathway with other cancer-related pathways, such as the Ras, EGFR, and redox pathways, indicate that simultaneous targeting of several key molecules in these pathways using either combination therapy or agents capable of modulating the functions of multiple targets will be required for effective cancer therapy. Our own experience in anticancer drug development demonstrated that genetic interactions can be exploited for the development of anticancer agents targeting multiple pathways and that performing robust compound optimization once a lead compound is identified is critical, as compounds with similar chemical structures and in vitro activity may have dramatically different *in vivo* toxicity and efficacy profiles.
